# Case Report: Improved Oxygenation after One Lung Ventilation in Severe Cardiomegaly due to Cor Pulmonale; analysis with Heart-Lung Interaction Approach

**DOI:** 10.12688/f1000research.171612.1

**Published:** 2026-01-05

**Authors:** Bambang Pujo Semedi, Willy Kurniawan, Arinanda Lalita Hayu, Yoppie Prim Avidar, Suryanti Chan

**Affiliations:** 1 Department of Anesthesiology and Reanimation, Airlangga University, Surabaya, East Java, Indonesia; 2 Department of Anesthesiology and Reanimation, Dr. Soetomo General Academic Hospital, Surabaya, East Java, Indonesia; 3 Universitas Dian Nuswantoro, Semarang, Central Java, Indonesia

**Keywords:** one-lung ventilation, cardiomegaly, thoracotomy.

## Abstract

**Introduction:**

One-lung ventilation (OLV) is used to isolate one lung during thoracic surgery, but manipulation and positioning can affect heart-lung interaction. Cardiomegaly may exacerbate these changes, especially in the left lateral decubitus (LLD) position.

**Objectives:**

To investigate the effect of cardiomegaly on heart-lung interaction during OLV, particularly in the LLD position.

**Case presentation:**

A 20-year-old male with recurrent spontaneous pneumothorax was scheduled for right-sided *bronchopleural* fistula repair via thoracotomy. The patient presented with cardiomegaly (cardiothoracic ratio 75%) and echocardiographic evidence of right ventricular and atrial dilation. In the LLD position, OLV led to desaturation when both lungs were ventilated, but oxygenation improved when only the left lung was ventilated.

**Results:**

Cardiomegaly alters heart-lung interaction during OLV, particularly in the LLD position. The enlarged heart exerts pressure on the left lung, impairing ventilation. When both lungs are ventilated in this position, ventilation is directed toward the right lung, reducing oxygenation and causing desaturation. However, restricting ventilation to the left lung improved oxygenation due to better lung compliance and less interference from the enlarged heart.

**Conclusions:**

Cardiomegaly affects heart-lung interaction during OLV in the LLD position. Oxygenation improves when only the left lung is ventilated, likely due to less compression of the left lung. The supine position may further enhance oxygenation even with bilateral ventilation. This case highlights the importance of considering cardiomegaly in OLV management. This section should be written as per the CARE checklist item 3.

## Introduction

One-lung ventilation (OLV) is a technique used during thoracotomy to selectively ventilate one lung while collapsing the other. This can be achieved using a double-lumen tube (DLT), a single-lumen tube with a bronchial blocker, or an endotracheal tube positioned endobronchially ( Butterworth et al., 2013). The procedure involves both manipulation of the lungs and changes in body positioning, which can affect heart-lung interaction. Non-ventilated but perfused lungs may result in a right-to-left shunt, a condition that can be partly mitigated by hypoxic pulmonary vasoconstriction and gravity, which redistributes blood flow to the lower lung ( Marongiu et al., 2020). However, OLV also affects cardiac function. Positive pressure ventilation can decrease venous return and systemic vascular resistance, while alveolar hypoxia may induce pulmonary vasoconstriction, increasing the workload on the right ventricle ( Slinger et al., 2019). These interactions between the heart and lungs are critical, as changes in one component often affect the other. In this case, we present a patient with cardiomegaly who experienced significant changes in oxygenation during OLV. The patient exhibited desaturation when both lungs were ventilated, but oxygenation improved when only the diseased lung was ventilated.

## Case report

**Patient information:** A 20-year-old male, weighing 45 kg with a height of 165 cm (BMI 16.5 kg/m ^2^), presented in March 2022 with sudden onset shortness of breath. He had no prior chronic illness until one year earlier, when he experienced moderate COVID-19 pneumonia. Since then, he reported reduced exercise tolerance and recurrent shortness of breath but had not sought medical care.

**Clinical findings:** On initial evaluation, he was alert, with blood pressure 100/70 mmHg, heart rate 110 bpm, respiratory rate 26–28 breaths per minute, oxygen saturation 95–96% on 2 L/min oxygen via nasal cannula, and temperature 36.7°C. Physical examination revealed decreased breath sounds on the right hemithorax with a thoracic drain in situ after recurrence.

### Timeline

Timeline of patient is presented by Table 1.

**Table 1.** Timeline of patient.

Date/Period	Event	Findings/Intervention	Outcome
March 2022	Sudden onset shortness of breath	Diagnosed with right spontaneous pneumothorax	Thoracic drain inserted
Day 8	Follow-up	Improvement on chest X-ray	Drain removed
Day 9	Recurrence of dyspnea	Repeat thoracic drain insertion	Symptom relief
Following days	Diagnostic imaging	CT scan → bronchopleural fistula (posterior segment, RUL)	Planned thoracotomy
Pre-op	Preoperative evaluation	Stable vitals; ABG: pH 7.37, PaO _2_ 80 mmHg, PaO _2_/FiO _2_ 200; Echo: RA/RV dilatation, pulmonary & tricuspid regurgitation	Intermediate probability of pulmonary hypertension
Intra-op	Induction & maintenance	Fentanyl, propofol, rocuronium; sevoflurane; double-lumen tube; VC ventilation	Stable at start
Intra-op (LLD, OLV)	Complication	Hypotension (75/45 mmHg), SpO _2_ ↓ to 88%	Norepinephrine & milrinone started; ventilator adjusted (TV 300 mL, RR 20, PEEP 8, FiO _2_ 100%) → SpO _2_ ↑ to 92%
Post-op	Supine, two-lung ventilation	Stable oxygenation	No further desaturation

**Diagnostic assessment:** The working diagnosis was a right *bronchopleural fistula* complicating spontaneous pneumothorax. The diagnosis was confirmed by thoracic computed tomography, while transthoracic echocardiography demonstrated right atrial and right ventricular dilatation with evidence of impaired cardiopulmonary reserve. Differential diagnoses, including persistent pneumothorax without fistula and interstitial lung disease, were considered but excluded based on clinical evaluation and imaging findings.

**Therapeutic intervention:** The patient underwent thoracotomy and bronchopleural fistula repair. Prior to induction, an arterial line was inserted, with baseline measurements showing a blood pressure of 105/55 mmHg, heart rate of 110 bpm, and oxygen saturation of 95% on 3 L/min of supplemental oxygen. Anesthesia was induced using fentanyl, propofol, and rocuronium, followed by endotracheal intubation with a 37 Fr left-sided double-lumen tube. Anesthesia was maintained with sevoflurane. Mechanical ventilation was initiated with a tidal volume of 360 mL, respiratory rate of 18 breaths per minute, PEEP of 5 cmH _2_O, and an inspired oxygen fraction of 0.5, resulting in an oxygen saturation of 99%. During one-lung ventilation in the left lateral decubitus position, the patient developed hypotension and oxygen desaturation. Vasopressor and inotropic support with norepinephrine (50 ng/kg/min) and milrinone (0.3 μg/kg/min) was initiated, and ventilatory parameters were adjusted accordingly, leading to partial hemodynamic stabilization and improvement in oxygenation.

**Follow-up and outcomes:** At the end of surgery, the patient was returned to the supine position with two-lung ventilation, after which oxygenation stabilized and no further desaturation occurred. Postoperative follow-up revealed stable respiratory function without recurrence of pneumothorax or desaturation events.

## Discussion

The incidence of hypoxemia during One-Lung Ventilation (OLV) has decreased significantly over time, from 25% in the 1970s to less than 10% today ( Semedi et al., 2021). The primary advantage of OLV is that it facilitates thoracic surgery by collapsing the lung on the operative side. However, this collapse often leads to a right-to-left intrapulmonary shunt, where blood from the collapsed, non-ventilated lung mixes with oxygenated blood from the ventilated lung. This can increase the PA-a O _2_ gradient (alveolar to arterial oxygen difference), potentially causing hypoxemia. Fortunately, hypoxic pulmonary vasoconstriction (HPV) reduces blood flow to the non-ventilated lung, helping to mitigate this effect ( Marongiu et al., 2020). However, in cases where atelectasis affects the dependent lung, oxygenation is further compromised due to V/Q mismatch (ventilation-perfusion mismatch) ( Rehatta et al., 2019).

In the present case, the patient exhibited cardiomegaly with dilation of both the right atrium (RA) and right ventricle (RV), along with an increased likelihood of pulmonary hypertension. This condition, common in patients with lung damage such as post-COVID-19 patients, can lead to pulmonary hypertension type 3 ( Taha et al., 2023). Pulmonary hypertension increases the workload of the RV, causing RV dilation and subsequently RA dilation. The elevated RV afterload due to increased pulmonary vascular resistance (PVR) further affects the patient’s hemodynamics.

Positive pressure ventilation during OLV can exacerbate these hemodynamic changes by increasing intrathoracic pressure, which in turn raises RA pressure and decreases venous return. This results in reduced RV preload and output, potentially worsening the patient’s condition. Additionally, excessive lung inflation can cause alveolar distension, compressing the alveolar vessels, thus increasing pulmonary vascular resistance and reducing cardiac output ( Guia et al., 2020). The use of fentanyl and propofol in this patient could further reduce cardiac function, contributing to systemic vasodilation. To manage these issues, norepinephrine was administered to raise systemic vascular resistance (SVR), while milrinone was used as an inotropic agent and pulmonary vasodilator to decrease RV afterload.

A particularly interesting phenomenon in this case occurred when the patient was positioned in the left lateral decubitus (LLD) position for surgery. In contrast to the typical pattern of hypoxemia observed during one-lung ventilation (OLV), this patient demonstrated improved oxygenation when only the dependent left lung was ventilated. Under usual circumstances, hypoxemia during OLV improves with two-lung ventilation; however, in this case, severe cardiomegaly with a cardiothoracic ratio of 75% altered the expected physiological response. The markedly enlarged heart exerted compressive forces on the dependent left lung in the LLD position, reducing lung compliance and impairing effective ventilation. Gravitational displacement of the mediastinum further exacerbated this effect, resulting in preferential airflow toward the non-dependent right lung during two-lung ventilation. Because the right lung was diseased, this redistribution of ventilation worsened ventilation–perfusion mismatch and contributed to hypoxemia, despite on-going perfusion of the dependent lung.

However, when the patient was ventilated with only the left lung, the positive pressure ventilation was effectively directed into the left lung, despite its suboptimal compliance due to heart compression. This resulted in improved ventilation-perfusion (V/Q) matching, which led to the resolution of hypoxemia. The finding is notable because it challenges the typical response seen in most patients undergoing OLV, where ventilation of both lungs typically results in better oxygenation.

After the operation, when the patient was returned to the supine position, two-lung ventilation no longer resulted in hypoxemia and oxygenation remained stable. In the supine position, posterior displacement of the heart reduces its compressive effect on the lungs, thereby improving lung expansion and ventilation. Nevertheless, cardiomegaly may still influence regional ventilation, particularly in the lower lobes, even in the supine position, as previously reported in the literature.

This case underscores the complexity of managing OLV in patients with cardiomegaly and pulmonary hypertension. The findings suggest that patient positioning, particularly in cases of significant heart enlargement, plays a crucial role in determining oxygenation outcomes during OLV. Further studies are needed to better understand the effects of cardiomegaly and pulmonary hypertension on heart-lung interactions during thoracic surgery.

## Conclusion

Severe cardiomegaly can affect the interaction between the heart and the lungs, particularly in the left lateral decubitus (LLD) position, where the enlarged heart can compress the left lung. This compression reduces lung compliance and disrupts the ventilation of the left lung, making it easier for positive pressure ventilation to move upward to inflate the right lung. Because there is inadequate ventilation and perfusion in the healthy left lung and the diseased right lung, a right-to-left shunt occurs, leading to hypoxemia. Surprisingly, One Lung Ventilation (OLV) in this case proves beneficial because positive pressure primarily directs air into the left lung. Despite the pressure exerted by the heart, this condition allows for ventilation along with perfusion, ultimately reducing the right-to-left shunt that causes hypoxemia.

## Consent

Written informed consent for the publication of this case report and any associated images has been obtained from the patient. The patient has given permission for their medical information to be published in this case report. All identifying information has been removed to ensure confidentiality, in accordance with ethical standards and privacy regulations.

## Data Availability

All data underlying the results are available as part of the article and no additional source data are required. The CARE checklist for this case report is available in the Zenodo repository, DOI: https://doi.org/10.5281/zenodo.18042365 ( Willy, K., 2025). Data are available under the terms of the Creative Commons Attribution 4.0 International license (CC-BY 4.0).
