# Case Report: Improved Oxygenation after One Lung Ventilation in Severe Cardiomegaly due to Cor Pulmonale; analysis with Heart-Lung Interaction Approach

**DOI:** 10.12688/f1000research.171612.3

**Published:** 2026-03-26

**Authors:** Bambang Pujo Semedi, Willy Kurniawan, Arinanda Lalita Hayu, Yoppie Prim Avidar, Suryanti Chan

**Affiliations:** 1 Department of Anesthesiology and Reanimation, Airlangga University, Surabaya, East Java, Indonesia; 2 Department of Anesthesiology and Reanimation, Dr. Soetomo General Academic Hospital, Surabaya, East Java, Indonesia; 3 Universitas Dian Nuswantoro, Semarang, Central Java, Indonesia

**Keywords:** one-lung ventilation, cardiomegaly, thoracotomy.

## Abstract

**Introduction:**

One-lung ventilation (OLV) is used to isolate one lung during thoracic surgery, but manipulation and positioning can affect heart-lung interaction. Cardiomegaly may exacerbate these changes, especially in the left lateral decubitus (LLD) position.

**Objectives:**

To investigate the effect of cardiomegaly on heart-lung interaction during OLV, particularly in the LLD position.

**Case presentation:**

A 20-year-old male with recurrent spontaneous pneumothorax was scheduled for right-sided *bronchopleural* fistula repair via thoracotomy. The patient presented with cardiomegaly (cardiothoracic ratio 75%) and echocardiographic evidence of right ventricular and atrial dilation. In the LLD position, OLV led to desaturation when both lungs were ventilated, but oxygenation improved when only the left lung was ventilated.

**Results:**

Cardiomegaly alters heart-lung interaction during OLV, particularly in the LLD position. The enlarged heart exerts pressure on the left lung, impairing ventilation. When both lungs are ventilated in this position, ventilation is directed toward the right lung, reducing oxygenation and causing desaturation. However, restricting ventilation to the left lung improved oxygenation due to better lung compliance and less interference from the enlarged heart.

**Conclusions:**

Cardiomegaly affects heart-lung interaction during OLV in the LLD position. Oxygenation improves when only the left lung is ventilated, likely due to less compression of the left lung. The supine position may further enhance oxygenation even with bilateral ventilation. This case highlights the importance of considering cardiomegaly in OLV management. This section should be written as per the CARE checklist item 3.

## Introduction

One-lung ventilation (OLV) is a technique used during thoracotomy to selectively ventilate one lung while collapsing the other. This can be achieved using a double-lumen tube (DLT), a single-lumen tube with a bronchial blocker, or an endotracheal tube positioned endobronchially ( Butterworth et al., 2013). Thoracic surgical procedures requiring lung isolation and lateral decubitus positioning may significantly influence heart–lung interactions. Non-ventilated but perfused lungs may result in a right-to-left shunt, a condition that can be partly mitigated by hypoxic pulmonary vasoconstriction and gravity, which redistributes blood flow to the lower lung ( Marongiu et al., 2020). However, OLV also affects cardiac function. Positive pressure ventilation can decrease venous return and systemic vascular resistance, while alveolar hypoxia may induce pulmonary vasoconstriction, increasing the workload on the right ventricle ( Slinger et al., 2019). These interactions between the heart and lungs are critical, as changes in one component often affect the other. In this case, we present a patient with cardiomegaly who experienced significant changes in oxygenation during OLV. Oxygen saturation remained stable during two-lung ventilation but decreased after the initiation of one-lung ventilation.

## Case report

**Patient information:** A 20-year-old male, weighing 45 kg with a height of 165 cm (BMI 16.5 kg/m ^2^), presented in March 2022 with sudden onset shortness of breath. He had no prior chronic illness until one year earlier, when he experienced moderate COVID-19 pneumonia. Since then, he reported reduced exercise tolerance and recurrent shortness of breath but had not sought medical care.

**Clinical findings:** On initial evaluation, he was alert, with blood pressure 100/70 mmHg, heart rate 110 bpm, respiratory rate 26–28 breaths per minute, oxygen saturation 95–96% on 2 L/min oxygen via nasal cannula, and temperature 36.7°C. Physical examination revealed decreased breath sounds on the right hemithorax with a thoracic drain in situ after recurrence. Baseline oxygenation was assessed using arterial blood gas analysis, which demonstrated a PaO _2_ of 80 mmHg with a PaO _2_/FiO _2_ ratio of approximately 200. The exact peripheral oxygen saturation (SpO _2_) on room air was not documented because the patient was already receiving supplemental oxygen due to respiratory symptoms at the time of evaluation.

### Timeline

Timeline of patient is presented by Table 1.

**Table 1.** Timeline of patient.

Date/Period	Event	Findings/Intervention	Outcome
March 2022	Sudden onset shortness of breath	Diagnosed with right spontaneous pneumothorax	Thoracic drain inserted
Day 8	Follow-up	Improvement on chest X-ray	Drain removed
Day 9	Recurrence of dyspnea	Repeat thoracic drain insertion	Symptom relief
Following days	Diagnostic imaging	CT scan → bronchopleural fistula (posterior segment, RUL)	Planned thoracotomy
Pre-op	Preoperative evaluation	Stable vitals; ABG: pH 7.37, PaO _2_ 80 mmHg, PaO _2_/FiO _2_ 200; Echo: RA/RV dilatation, pulmonary & tricuspid regurgitation	Intermediate probability of pulmonary hypertension
Intra-op	Induction & intubation	Fentanyl, propofol, rocuronium; sevoflurane; double-lumen tube; VC ventilation	Hemodynamically stable
Intra-op	Positioning	Patient positioned in left lateral decubitus (LLD); two-lung ventilation maintained	No immediate desaturation
Intra-op	Initiation of OLV	One-lung ventilation of left lung initiated	Oxygen saturation began to decrease
Intra-op	Hemodynamic event	Hypotension (75/45 mmHg) and SpO _2_ decreased to 88%	Suspected RV strain
Intra-op	Management	Norepinephrine and milrinone started; ventilator adjusted (TV 300 mL, RR 20, PEEP 8, FiO _2_ 100%)	SpO _2_ improved to 92%
Post-op	Supine, two-lung ventilation	Oxygenation stabilized	No further desaturation

**Diagnostic assessment:** The working diagnosis was a right bronchopleural fistula complicating spontaneous pneumothorax. The diagnosis was confirmed by thoracic computed tomography. Transthoracic echocardiography demonstrated right atrial and right ventricular dilatation with moderate tricuspid regurgitation. The right ventricular systolic pressure (RVSP) could not be quantified from the available echocardiographic report **.** Differential diagnoses, including persistent pneumothorax without fistula and interstitial lung disease, were considered but excluded based on clinical evaluation and imaging findings.

**Therapeutic intervention:** The patient was transferred to the operating room with standard ASA monitoring. Prior to induction, an arterial line was inserted for continuous blood pressure monitoring. Baseline measurements showed blood pressure 105/55 mmHg, heart rate 110 bpm, and oxygen saturation 95% on 3 L/min oxygen via nasal cannula.

Anesthesia was induced with fentanyl, propofol, and rocuronium. After adequate muscle relaxation, a 37 Fr left-sided double-lumen tube (DLT) was inserted. Correct placement was confirmed clinically by auscultation and selective lung ventilation assessment. Following intubation, mechanical ventilation was initiated in volume-controlled mode with a tidal volume of 360 mL, respiratory rate 18 breaths per minute, PEEP 5 cmH _2_O, and FiO _2_ 0.5. Oxygen saturation improved to 99%, and hemodynamics remained stable.

After surgical preparation, the patient was positioned in the left lateral decubitus (LLD) position. Two-lung ventilation was initially maintained during positioning, and no immediate desaturation occurred.

One-lung ventilation (OLV) of the left lung was then initiated to facilitate right thoracotomy. Shortly after initiation of OLV in the LLD position, the patient developed progressive hypotension (blood pressure decreased to 75/45 mmHg) accompanied by oxygen desaturation to 88%. At this time, ventilatory parameters were reassessed and adjusted. Tidal volume was reduced to 300 mL, respiratory rate increased to 20 breaths per minute, PEEP increased to 8 cmH _2_O, and FiO _2_ increased to 1.0. Tidal volume was reduced to decrease airway pressure and minimize air leakage through the bronchopleural fistula. To maintain adequate minute ventilation, the respiratory rate was increased. This strategy also helped limit excessive intrathoracic pressure that could worsen hemodynamic compromise.

Vasopressor and inotropic support were initiated with norepinephrine (50 ng/kg/min) and milrinone (0.3 μg/kg/min) to address suspected right ventricular strain and reduced systemic vascular resistance. Following these interventions, oxygen saturation improved gradually to 92%, and blood pressure stabilized.

No major surgical traction or compression events were noted at the time of desaturation. The remainder of the procedure proceeded under careful hemodynamic and ventilatory monitoring.

At the end of surgery, the patient was returned to the supine position and two-lung ventilation was resumed. Oxygenation stabilized, and no further desaturation episodes were observed.

**Follow-up and outcomes:** At the end of surgery, the patient was returned to the supine position with two-lung ventilation, after which oxygenation stabilized and no further desaturation occurred. Postoperative follow-up revealed stable respiratory function without recurrence of pneumothorax or desaturation events.

## Discussion

In this case, intraoperative desaturation occurred shortly after initiation of one-lung ventilation (OLV) in the left lateral decubitus (LLD) position, accompanied by hypotension. Several potential mechanisms were considered, including double-lumen tube (DLT) malposition, increased right-to-left intrapulmonary shunt during OLV, atelectasis of the dependent lung, acute right ventricular dysfunction, and mechanical compression of the lung due to severe cardiomegaly.

Several mechanisms may explain the observed cardiopulmonary interaction in this case. One-lung ventilation can increase pulmonary vascular resistance due to hypoxic pulmonary vasoconstriction and altered pulmonary blood flow distribution. In patients with pre-existing cardiomegaly and limited cardiopulmonary reserve, this increase in pulmonary vascular resistance may elevate right ventricular afterload and impair right ventricular output. Furthermore, changes in ventilation–perfusion relationships during lung isolation may worsen gas exchange and contribute to intraoperative oxygen desaturation.

DLT malposition was considered unlikely based on clinical reassessment and selective ventilation findings. The close temporal relationship between positioning, initiation of OLV, hemodynamic instability, and oxygen desaturation suggested a combined cardiopulmonary interaction rather than an isolated ventilatory issue.

The incidence of hypoxemia during OLV has decreased significantly over time, from approximately 25% in the 1970s to less than 10% in modern practice ( Semedi et al., 2021). Under normal circumstances, hypoxemia during OLV is primarily caused by right-to-left intrapulmonary shunting from the non-ventilated lung. Hypoxic pulmonary vasoconstriction (HPV) helps mitigate this effect by reducing perfusion to the collapsed lung and redistributing blood flow to the ventilated lung ( Marongiu et al., 2020). However, if atelectasis affects the dependent lung, oxygenation may worsen due to ventilation–perfusion (V/Q) mismatch ( Rehatta et al., 2019). Typically, oxygenation improves with two-lung ventilation.

The present case deviated from this classical pattern. The patient had severe cardiomegaly (cardiothoracic ratio 75%) with right atrial (RA) and right ventricular (RV) dilation and suspected pulmonary hypertension. Pulmonary hypertension, which may develop in patients with chronic lung disease or post-COVID-19 pulmonary sequelae, increases RV afterload and reduces cardiopulmonary reserve ( Taha et al., 2023). Elevated pulmonary vascular resistance (PVR) increases RV workload and may impair cardiac output, especially under stress conditions.

Positive pressure ventilation during OLV can further exacerbate these hemodynamic alterations. Increased intrathoracic pressure may reduce venous return and elevate right atrial pressure, thereby compromising RV preload. In addition, excessive alveolar distension may compress pulmonary capillaries and increase PVR, potentially worsening RV afterload and decreasing cardiac output ( Guia et al., 2020). Positive pressure ventilation may also influence systemic vascular resistance through changes in autonomic tone and intrathoracic pressure dynamics. A reduction in venous return and cardiac output may trigger compensatory vasodilatory responses or altered sympathetic activity, which in certain circumstances can contribute to decreased systemic vascular resistance ( Corp, A. et al., 2021). The observed intraoperative hypotension supports the possibility of transient RV compromise in this setting. Administration of norepinephrine and milrinone was therefore aimed at increasing systemic vascular resistance and reducing RV afterload.

The presence of a bronchopleural fistula may also influence intrathoracic pressure dynamics during mechanical ventilation. During two-lung ventilation, the fistula may partially limit excessive increases in intrathoracic pressure by allowing air leakage. However, during one-lung ventilation, the resulting increase in pulmonary vascular resistance may significantly elevate right ventricular afterload. In patients with impaired right ventricular reserve, this may compromise right ventricular output and contribute to hemodynamic instability.

A distinctive feature of this case was the improvement in oxygenation when only the dependent left lung was ventilated in the LLD position. We hypothesize that severe cardiomegaly altered the usual distribution of ventilation. In the LLD position, gravitational displacement of the mediastinum combined with marked cardiac enlargement may have exerted compressive forces on the dependent left lung, reducing its compliance during two-lung ventilation. This could have redirected ventilation preferentially toward the non-dependent right lung, which was diseased, thereby worsening V/Q mismatch despite ongoing perfusion of the dependent lung.

After returning the patient to the supine position and resuming two-lung ventilation, oxygenation stabilized. In the supine position, posterior displacement of the enlarged heart may reduce its compressive effect on lung parenchyma, thereby improving regional ventilation. Nonetheless, cardiomegaly may still influence lower-lobe ventilation distribution even in the supine position.

This case suggests that in patients with severe cardiomegaly and suspected pulmonary hypertension, oxygenation behavior during OLV may not follow classical physiological expectations. Unexpected hypoxemia in such patients should prompt evaluation not only of airway devices and ventilatory settings, but also of positional and mediastinal effects on lung mechanics. Awareness of altered heart–lung interaction may help guide intraoperative decision-making and prevent unnecessary escalation of interventions.

### Limitations

This case report has several limitations. Detailed serial intraoperative physiological parameters such as dynamic lung compliance, airway pressure trends, serial arterial blood gas values, and comprehensive echocardiographic indices were not systematically documented, as this case was not initially managed as a prospective physiological study. In addition, the right ventricular systolic pressure (RVSP) could not be quantified from the available echocardiographic report, limiting precise assessment of pulmonary hypertension severity. Therefore, the proposed mechanisms remain inferential and based on available clinical data combined with established heart–lung interaction principles. Future prospective studies incorporating advanced hemodynamic and respiratory monitoring would be valuable to confirm these observations.

## Conclusion

This case highlights the complex cardiopulmonary interactions that may occur during one-lung ventilation in patients with impaired cardiopulmonary reserve. Careful anticipation of potential hemodynamic changes and appropriate adjustment of ventilatory parameters are essential to maintain oxygenation and hemodynamic stability in such patients.

## Consent

Written informed consent for the publication of this case report and any associated images has been obtained from the patient. The patient has given permission for their medical information to be published in this case report. All identifying information has been removed to ensure confidentiality, in accordance with ethical standards and privacy regulations.

## Data Availability

All data underlying the results are available as part of the article and no additional source data are required. The CARE checklist for this case report is available in the Zenodo repository, DOI: https://doi.org/10.5281/zenodo.18042365 ( Willy, K., 2025). Data are available under the terms of the Creative Commons Attribution 4.0 International license (CC-BY 4.0).
