# Supplementary material for: Case Report: Improved Oxygenation after One Lung Ventilation in Severe Cardiomegaly due to Cor Pulmonale; analysis with Heart-Lung Interaction Approach
Source: F1000Res. 2026 Apr 19;15:2. Originally published 2026 Jan 5. [Version 4] doi: 10.12688/f1000research.171612.4 (PMC13126001; doi:10.12688/f1000research.171612.4)
Supplement: Peer review history

# Reviewer report: Reviewer response for version 4

**DOI:** 10.5256/f1000research.198647.r476410

**Published:** 2026-04-28

**Authors:** Zhiyao Wang

**Affiliations:** 1 Fudan University, Shanghai, China

The authors have sufficiently addressed all comments and concerns.

Are enough details provided of any physical examination and diagnostic tests, treatment given and outcomes?

Partly

Is the case presented with sufficient detail to be useful for other practitioners?

Partly

Is sufficient discussion included of the importance of the findings and their relevance to future understanding of disease processes, diagnosis or treatment?

Partly

Is the background of the case’s history and progression described in sufficient detail?

Partly

Reviewer Expertise:

Anesthesiology; perioperative analgesia; pain neuroscience; neuroimmune mechanisms;

I confirm that I have read this submission and believe that I have an appropriate level of expertise to confirm that it is of an acceptable scientific standard.

# Reviewer report: Reviewer response for version 4

**DOI:** 10.5256/f1000research.198647.r476409

**Published:** 2026-04-22

**Authors:** Praveen Kumar Neema

**Affiliations:** 1 Amrita Institute of Medical Sciences, Kochi, Kerala, India

The authors have revised the manuscript and is now acceptable.

Are enough details provided of any physical examination and diagnostic tests, treatment given and outcomes?

Yes

Is the case presented with sufficient detail to be useful for other practitioners?

Partly

Is sufficient discussion included of the importance of the findings and their relevance to future understanding of disease processes, diagnosis or treatment?

No

Is the background of the case’s history and progression described in sufficient detail?

Partly

Reviewer Expertise:

Cardiovascular physiology; cardiopulmonary bypass, adult and pediatric cardiac anesthesia, thoracic anesthesia,

I confirm that I have read this submission and believe that I have an appropriate level of expertise to confirm that it is of an acceptable scientific standard.

# Reviewer report: Reviewer response for version 3

**DOI:** 10.5256/f1000research.197689.r470938

**Published:** 2026-04-08

**Authors:** Praveen Kumar Neema

**Affiliations:** 1 Amrita Institute of Medical Sciences, Kochi, Kerala, India

2nd revision

 Introduction

 Change 'lungs' to lung

 Paragraph 

 After surgical preparation, the patient was positioned in the left lateral decubitus (LLD) position. Two-lung ventilation

 was initially maintained during positioning, and no immediate desaturation occurred. 

**To the above paragraph add the sentence** 

 During two-lung ventilation, an unknown portion of tidal volume was leaking through the bronchopleural fistula.

 One-lung ventilation (OLV) of the left lung was then initiated to facilitate right thoracotomy. Shortly after initiation of OLV in the LLD position, the patient developed progressive hypotension (blood pressure decreased to 75/45 mmHg) accompanied by oxygen desaturation to 88%. At this time, ventilatory parameters were reassessed and adjusted. Tidal volume was reduced to 300 mL, respiratory rate increased to 20 breaths per minute, PEEP increased to 8 cmH2O, and

 FiO2 increased to 1.0. Tidal volume was reduced to decrease airway pressure and minimize air leakage through the

 bronchopleural fistula. To maintain adequate minute ventilation, the respiratory rate was increased. This strategy also helped limit excessive intrathoracic pressure that could worsen hemodynamic compromise.

**Modify the paragraph as**

 One-lung ventilation (OLV) of the left lung was then initiated to facilitate right thoracotomy. Shortly after initiation of

 OLV in the LLD position, the patient developed progressive hypotension (blood pressure decreased to 75/45 mmHg)

 accompanied by oxygen desaturation to 88%. At this time, the volume loss through the bronchopleural fistula stopped; therefore, the ventilatory parameters were reassessed and adjusted. Tidal volume was reduced to 300 mL, respiratory rate increased to 20 breaths per minute, PEEP increased to 8 cmH2O, and FiO2 increased to 1.0. Tidal volume was reduced to decrease airway pressure. This strategy helped limit excessive intrathoracic pressure that could worsen hemodynamic compromise. 

**Rewrite the paragraph**

 A distinctive feature of this case was the improvement in oxygenation when only the dependent left lung was ventilated in the LLD position. We hypothesize that severe cardiomegaly altered the usual distribution of ventilation. In the LLD position, gravitational displacement of the mediastinum combined with marked cardiac enlargement may have exerted compressive forces on the dependent left lung, reducing its compliance during two-lung ventilation. This could have redirected ventilation preferentially toward the non-dependent right lung, which was diseased, thereby worsening V/Q mismatch despite ongoing perfusion of the dependent lung.

 A distinctive feature of this case was better oxygenation when both the lungs were ventilated in the LLD position. We hypothesize that during one lung ventilation, severe cardiomegaly and increased intrathoracic pressure increased the compression of pulmonary vasculature and aggravated right ventricular dysfunction. This could have

 redirected perfusion toward the non-dependent right lung, which was diseased, thereby worsening V/Q mismatch. Conceivably, reduced tidal volume, milrinone and nor-epinephrine combined together improved RV performance, increased the systemic arterial pressure and arterial saturation.

Are enough details provided of any physical examination and diagnostic tests, treatment given and outcomes?

Yes

Is the case presented with sufficient detail to be useful for other practitioners?

Partly

Is sufficient discussion included of the importance of the findings and their relevance to future understanding of disease processes, diagnosis or treatment?

No

Is the background of the case’s history and progression described in sufficient detail?

Partly

Reviewer Expertise:

Cardiovascular physiology; cardiopulmonary bypass, adult and pediatric cardiac anesthesia, thoracic anesthesia,

I confirm that I have read this submission and believe that I have an appropriate level of expertise to confirm that it is of an acceptable scientific standard, however I have significant reservations, as outlined above.

# Response

**Published:** 2026-04-09

**Authors:** Suryanti Suryanti

**Affiliations:** Faculty of Medicine, Lincoln University College; Universitas Dian NUswantoro, Semarang, Indonesia

Dear Reviewer,

 Thank you very much for your careful review and valuable comments on our manuscript. We have revised the manuscript accordingly and addressed each point as follows:

1. **Introduction**
  - Comment: Change “lungs” to “lung”
  - Response: Revised as suggested.
2. **Paragraph on patient positioning and two-lung ventilation**
  - Comment: Add the sentence regarding tidal volume leakage through the bronchopleural fistula
  - Response: The sentence “During two-lung ventilation, an unknown portion of tidal volume was leaking through the bronchopleural fistula.” has been added to clarify the presence of air leakage during two-lung ventilation.
3. **Paragraph on one-lung ventilation (OLV) and ventilatory adjustments**
  - Comment: Modify the paragraph as suggested
  - Response: The paragraph has been revised accordingly. We have clarified that volume loss through the bronchopleural fistula ceased after initiation of OLV and adjusted the explanation of ventilatory management to focus on reduction of airway pressure and limitation of intrathoracic pressure to mitigate hemodynamic compromise.
4. **Discussion paragraph (physiological interpretation)**
  - Comment: Rewrite the paragraph
  - Response: The paragraph has been substantially revised as suggested. We have corrected the interpretation to reflect improved oxygenation during two-lung ventilation rather than OLV. The discussion now emphasizes the role of severe cardiomegaly, increased intrathoracic pressure, pulmonary vascular compression, and right ventricular dysfunction in worsening ventilation–perfusion mismatch. We also incorporated the contribution of reduced tidal volume, milrinone, and norepinephrine in improving right ventricular performance and systemic oxygenation.

We believe these revisions have significantly improved the clarity and physiological consistency of the manuscript. We sincerely appreciate your insightful comments.

 Kind regards,

 On behalf of all authors,

 Suryanti Chan

# Reviewer report: Reviewer response for version 2

**DOI:** 10.5256/f1000research.196561.r461303

**Published:** 2026-03-12

**Authors:** Praveen Kumar Neema

**Affiliations:** 1 Amrita Institute of Medical Sciences, Kochi, Kerala, India

The sentence, “The procedure involves both manipulation of the lungs and changes in body positioning, which can affect heart-lung interaction” is not clear, do the authors mean ‘surgical procedure’ please rephrase

 In view of continuous oxygen requirement, did you measure peripheral oxygen saturation (SpO2) on room air?

 However, OLV also affects cardiac function. Positive pressure ventilation can decrease venous return and systemic vascular resistance. How do you explain a decrease in systemic vascular resistance with positive pressure ventilation? please expand and provide reference.

 The patient exhibited desaturation when both lungs were ventilated, but oxygenation improved when only the diseased lung was ventilated. The timeline described in the case report and the sentence do not match; as per the case report, the saturation was normal when both the lungs were ventilated and the patient developed desaturation after the initiation of one lung ventilation.

 Transthoracic echocardiography demonstrated right atrial and right ventricular dilatation with evidence of impaired cardiopulmonary reserve, what was the right ventricular systolic pressure (RVSP) measured by TR jet?

 Following intubation, mechanical ventilation was initiated in volume-controlled mode with a tidal volume of 360 mL, respiratory rate 18 breaths per minute, PEEP 5cmH2O, and FiO2 0.5. Oxygen saturation improved to 99%, and hemodynamics remained stable. Do you mean two lung ventilation? How did you manage loss of tidal volume through bronchopleural fistula?

 Table 1: comment on right ventricular systolic pressure

 Timeline-Intraop: describe ventilation parameters in detail. From the timeline it is not clear when the patient deteriorated and developed desaturation and hypotension. In the case report it is mentioned that initially two-lung ventilation was maintained and there was no desaturation. Does it mean that patient developed complications after initiating one lung ventilation?   

 Tidal volume was reduced to 300 mL, respiratory rate increased to 20 breaths per minute, PEEP increased to 8cmH2O, and FiO2 increased to 1.0. what was the rationale?

 The close temporal relationship between positioning, initiation of OLV, hemodynamic instability, and oxygen desaturation suggested a combined cardiopulmonary interaction rather than an isolated ventilatory issue, please expand. What were the possible mechanisms and how did you assess them and finally what was the responsible mechanism?

 The hypothesis, ‘The present case deviated from this classical pattern. The patient had severe cardiomegaly…and increase PVR, potentially worsening RV afterload and decreasing cardiac output (Guia et al., 2020). The observed intraoperative hypotension supports the possibility of transient RV compromise in this setting. You should discuss the role of bronchopleural fistula during two lung ventilation, I believe, during two lung ventilation, the bronchopleural fistula prevented excessive increase in intrathoracic pressure and pulmonary vascular resistance. With the initiation of OLV, the pulmonary vascular resistance severely, which increased tricuspid and pulmonary regurgitation and severely compromised right ventricular cardiac output. Consequently, decreasing left ventricular cardiac output and precipitating severe hypotension and desaturation. The vicious cycle was interrupted once tidal volume was decreased. The decrease in right ventricular cardiac output is expected to be pronounced in presence of right ventricular dysfunction.

 We hypothesize…thereby worsening V/Q mismatch despite ongoing perfusion of the dependent lung; this is possible, if there is no bronchopleural fistula; however, the effect is expected to be mild in presence of bronchopleural fistula.

 When OLV was instituted…explanation is physiologically consistent with known heart–lung interaction principles (Marongiu et al., 2020). This paragraph can be removed

 This case suggests that in patients with severe cardiomegaly and suspected pulmonary hypertension, oxygenation behavior during OLV may not follow classical physiological expectations, I do not agree with the statement. The deterioration of hemodynamic and saturation should be anticipated and, perhaps, appropriate adjustments of ventilation parameters would have prevented the deterioration.

Are enough details provided of any physical examination and diagnostic tests, treatment given and outcomes?

Yes

Is the case presented with sufficient detail to be useful for other practitioners?

Partly

Is sufficient discussion included of the importance of the findings and their relevance to future understanding of disease processes, diagnosis or treatment?

No

Is the background of the case’s history and progression described in sufficient detail?

Partly

Reviewer Expertise:

Cardiovascular physiology; cardiopulmonary bypass, adult and pediatric cardiac anesthesia, thoracic anesthesia,

I confirm that I have read this submission and believe that I have an appropriate level of expertise to confirm that it is of an acceptable scientific standard, however I have significant reservations, as outlined above.

# Response

**Published:** 2026-03-12

**Authors:** Suryanti Suryanti

**Affiliations:** Faculty of Medicine, Lincoln University College; Universitas Dian NUswantoro, Semarang, Indonesia

We sincerely thank the reviewer for the insightful and constructive comments. We have carefully revised the manuscript to address all concerns. Detailed responses to each comment are provided below, and the corresponding revisions have been incorporated into the manuscript.

 1. **Comment 1**: The sentence, “The procedure involves both manipulation of the lungs and changes in body positioning, which can affect heart-lung interaction” is not clear, do the authors mean ‘surgical procedure’ please rephrase.

**Response: **Thank you for this suggestion. The sentence referred to the thoracic surgical procedure requiring one-lung ventilation and lateral positioning. We have revised the sentence to clarify this meaning. **Revision in manuscript: **The sentence has been revised as follows: “Thoracic surgical procedures requiring lung isolation and lateral decubitus positioning may significantly influence heart–lung interactions.”

 2. **Comment 2**: In view of continuous oxygen requirement, did you measure peripheral oxygen saturation (SpO₂) on room air?

**Response: **Thank you for this important question. Unfortunately, the exact SpO₂ value on room air was not documented in the medical record because the patient was already receiving supplemental oxygen due to respiratory symptoms at the time of evaluation. Baseline oxygenation was assessed using arterial blood gas analysis, which showed PaO₂ 80 mmHg with a PaO₂/FiO₂ ratio of approximately 200. We have clarified this point in the revised manuscript.

**Revision in manuscript: **A clarifying sentence has been added in the case presentation section indicating that baseline oxygenation was assessed using arterial blood gas analysis.

 3. **Comment 3**: However, OLV also affects cardiac function. Positive pressure ventilation can decrease venous return and systemic vascular resistance. How do you explain a decrease in systemic vascular resistance with positive pressure ventilation? Please expand and provide reference.

**Response: **Thank you for this important comment. We have expanded the discussion to clarify the hemodynamic effects of positive pressure ventilation. Positive pressure ventilation increases intrathoracic pressure, which reduces venous return and may decrease left ventricular preload. Additionally, reduced preload and cardiac output may lead to reflex vasodilation or altered autonomic tone, contributing to decreased systemic vascular resistance in some clinical settings. Relevant references have been added to support this explanation. **Revision in manuscript: **The discussion section has been expanded to explain the hemodynamic effects of positive pressure ventilation on venous return, cardiac output, and systemic vascular resistance, with additional references.

 4. **Comment 4:** The patient exhibited desaturation when both lungs were ventilated, but oxygenation improved when only the diseased lung was ventilated. The timeline described in the case report and the sentence do not match.

**Response: **We appreciate the reviewer’s careful observation. The reviewer is correct that the description was inconsistent with the timeline. The sentence has been corrected to reflect the actual sequence of events. **Revision in manuscript: **The corrected sentence is: “Oxygen saturation remained stable during two-lung ventilation but decreased after the initiation of one-lung ventilation.”

 5. **Comment 5**: Transthoracic echocardiography demonstrated right atrial and right ventricular dilatation… what was the right ventricular systolic pressure (RVSP) measured by TR jet?

**Response**: Thank you for this important comment. Unfortunately, the exact right ventricular systolic pressure (RVSP) derived from the tricuspid regurgitation jet was not documented in the available echocardiography report in the medical record. However, transthoracic echocardiography demonstrated right atrial and right ventricular dilatation with moderate tricuspid regurgitation, suggesting impaired right heart function and possible elevation of right-sided pressures. We have clarified this point in the revised manuscript. **Revision in manuscript**: A clarifying sentence has been added in the echocardiography findings section indicating that the RVSP value was not available in the echocardiography report.

 6. **Comment 6: **Do you mean two lung ventilation? How did you manage loss of tidal volume through bronchopleural fistula?

**Response: **Yes, the reviewer is correct that the ventilation described refers to two-lung ventilation following intubation. This has been clarified in the revised manuscript. Regarding tidal volume loss through the bronchopleural fistula, ventilation was carefully monitored and adjusted to minimize air leakage while maintaining adequate oxygenation. Lung isolation and ventilator parameter adjustments were used to reduce leakage and optimize ventilation. **Revision in manuscript: **The text has been revised to clarify that two-lung ventilation was initially used and to briefly describe the strategy for managing tidal volume loss due to the bronchopleural fistula.

 7. **Comment 7**: Table 1: comment on right ventricular systolic pressure.

**Response**: Thank you for this suggestion. Unfortunately, the right ventricular systolic pressure (RVSP) derived from the tricuspid regurgitation jet was not documented in the available echocardiography report in the medical record. Therefore, the RVSP value could not be included in Table 1. We have clarified this limitation in the revised manuscript. **Revision in manuscript: **A clarifying note has been added in the echocardiography findings section indicating that the RVSP value was not available in the echocardiography report.

 8. **Comment 8: **Timeline – intraoperative events are unclear.

**Response: **Thank you for pointing this out. The intraoperative timeline has been revised to clarify the sequence of ventilation strategies, oxygen desaturation, and hemodynamic changes.

**Revision in manuscript: **The timeline figure and corresponding text have been updated to clearly indicate:

- initiation of two-lung ventilation
- transition to one-lung ventilation
- onset of desaturation and hypotension
- subsequent ventilatory adjustment

9. **Comment 9: **Tidal volume was reduced to 300 mL… what was the rationale?

**Response: **The rationale was to reduce airway pressure and minimize air leakage through the bronchopleural fistula while maintaining adequate ventilation. Lower tidal volume with increased respiratory rate was used to maintain minute ventilation and reduce intrathoracic pressure.

**Revision in manuscript: **This rationale has been added to the intraoperative management section.

 10. **Comment 10:** Please expand possible mechanisms explaining the cardiopulmonary interaction.

**Response:**

 We thank the reviewer for this important suggestion. The discussion has been expanded to describe possible mechanisms including:

- increased pulmonary vascular resistance during OLV
- right ventricular afterload elevation
- impaired right ventricular output in the presence of cardiomegaly
- altered ventilation-perfusion relationships

These mechanisms were considered in explaining the observed intraoperative hemodynamic instability.

**Revision in manuscript: **Additional discussion has been included in the Discussion section.

 11. **Comment 11: **hypothesis regarding bronchopleural fistula and RV failure

**Response: **We appreciate this insightful physiological interpretation. We agree that the bronchopleural fistula may have influenced intrathoracic pressure dynamics during two-lung ventilation. We have incorporated this explanation into the discussion and acknowledged that increased pulmonary vascular resistance during OLV may have contributed to right ventricular compromise in this patient.

**Revision in manuscript: **The discussion now includes a paragraph discussing the potential role of bronchopleural fistula in modulating intrathoracic pressure and pulmonary vascular resistance during ventilation.

 12. **Comment 12: **Paragraph beginning “When OLV was instituted…” can be removed.

**Response: **Thank you for this suggestion. As recommended, the paragraph has been removed to improve clarity and avoid redundancy.

 13. **Comment 13:** “I do not agree with the statement…”

**Response: **We appreciate the reviewer’s perspective. We have revised the conclusion to avoid overstating the finding and to emphasize that careful anticipation and adjustment of ventilatory parameters are important in patients with cardiomegaly undergoing OLV. **Revision in manuscript:**

 The conclusion has been modified to reflect a more cautious interpretation of the findings.

# Reviewer report: Reviewer response for version 1

**DOI:** 10.5256/f1000research.189236.r452227

**Published:** 2026-02-12

**Authors:** Zhiyao Wang

**Affiliations:** 1 Fudan University, Shanghai, China

The background and progress are described to some extent, but not sufficiently:

 1) The overall timeline is unclear;

 2) There is a lack of description of the preoperative respiratory status;

 3) Descriptions of key events are still lacking, such as the specific timing of changes in body position, when one-lung ventilation was started, how the DLT position was confirmed, whether there was surgical traction/compression, re-expansion procedures, changes in ABG, changes in airway pressure/compliance, etc. The text lacks physical examination-related content such as dynamic changes in vital signs, respiratory examination, signs of respiratory distress, and cardiac examination; in terms of diagnostic examinations, it lacks indicators reflecting the severity of cardiopulmonary function, such as key echocardiographic parameters (TR Vmax, estimated PASP, TAPSE), right ventricular function, estimated pulmonary artery pressure, baseline hemodynamics, and exercise tolerance; follow-up is too brief, lacking details on objective endpoints such as postoperative imaging re-examination, ABG/echocardiography, chest tube indwelling time, whether the condition relapsed, and length of hospital stay. The discussion section still has shortcomings.

 First, the hypotheses should be discussed based on key objective physiological data (vital signs, ABG, airway pressure/compliance, EtCO₂ trends, etc.). Second, it is recommended to discuss the reasons for the intraoperative oxygen saturation decrease and the basis for exclusion. Finally, it is recommended to elaborate on the clinical significance of this case. Due to the insufficient detail in the case description, it is currently not sufficient to provide guidance for other clinicians in real-world clinical scenarios.

Are enough details provided of any physical examination and diagnostic tests, treatment given and outcomes?

Partly

Is the case presented with sufficient detail to be useful for other practitioners?

Partly

Is sufficient discussion included of the importance of the findings and their relevance to future understanding of disease processes, diagnosis or treatment?

Partly

Is the background of the case’s history and progression described in sufficient detail?

Partly

Reviewer Expertise:

clinical anesthesia; acute pain; chronic pain

I confirm that I have read this submission and believe that I have an appropriate level of expertise to confirm that it is of an acceptable scientific standard, however I have significant reservations, as outlined above.

# Response

**Published:** 2026-02-12

**Authors:** Suryanti Suryanti

**Affiliations:** Faculty of Medicine, Lincoln University College; Universitas Dian NUswantoro, Semarang, Indonesia

We sincerely thank the reviewer for the detailed and insightful comments. We fully agree that additional physiological parameters such as serial ABG measurements, detailed airway pressure/compliance trends, comprehensive echocardiographic indices, and extended follow-up data would strengthen the scientific rigor of this report.

 However, as this case was retrospectively prepared from routine clinical practice rather than a prospectively designed physiological study, certain detailed intraoperative parameters were not systematically recorded or are no longer retrievable from the medical record.

 Nevertheless, we have carefully revised the manuscript to:

1. Clarify the intraoperative timeline and sequence of events.
2. Expand the description of preoperative cardiopulmonary status based on available data.
3. Provide a more structured physiological interpretation based on the documented vital signs and clinical course.
4. Include a dedicated “Limitations” paragraph in the Discussion acknowledging the absence of detailed serial physiological data.
5. Emphasize that this report is hypothesis-generating and aims to highlight a potentially under-recognized heart–lung interaction phenomenon in patients with severe cardiomegaly undergoing OLV.

We agree that future prospective studies with comprehensive hemodynamic and respiratory monitoring would be necessary to validate the proposed mechanisms.

 We sincerely appreciate the reviewer’s comments, which have helped us improve the clarity and transparency of our report.
